# Cross talk between serum Kisspeptin-Leptin during assisted reproduction techniques

**DOI:** 10.12669/pjms.342.14078

**Published:** 2018

**Authors:** Rehana Rehman, Zehra Jamil, Aqsa Khalid, Syeda Sadia Fatima

**Affiliations:** 1 Rehana Rehman, Department of Biological and Biomedical Sciences, Aga Khan University, Stadium Road, Karachi, Pakistan; 2 Zehra Jamil, Department of Biological and Biomedical Sciences, Aga Khan University, Stadium Road, Karachi, Pakistan; 3 Aqsa Khalid, Department of Biological and Biomedical Sciences, Aga Khan University, Stadium Road, Karachi, Pakistan; 4 Syeda Sadia Fatima, Department of Biological and Biomedical Sciences, Aga Khan University, Stadium Road, Karachi, Pakistan

**Keywords:** Assisted Reproductive techniques, Kisspeptin, Leptin, Body Mass index

## Abstract

**Background & objective:**

Leptin facilitates onset of puberty by impact on hypothalamic Kisspeptin, gonadotropin releasing hormone, follicle stimulating and luteinizing hormone. The link of peripheral Leptin-Kisspeptin in regulating the ovarian and endometrial tissue in relation to adiposity is unknown. Therefore, we wanted to identify Kisspeptin-Leptin association with body mass index (BMI) and success of assisted reproductive treatments (ART) in infertile females.

**Methods:**

A cross sectional study was carried from August 2014 till May 2016 after receiving ethical approval at Australian Concept Infertility Medical Centre, and Aga Khan University. The study group comprised of females with an age range of 25-37 year who had duration of unexplained infertility for more than two years. They were grouped as; underweight (<18 kg/m^2^), normal weight (18-22.9 kg/m^2^), overweight 23-24.99 kg/m^2^ and obese (>25 kg/m^2^). Kisspeptin and Leptin levels were measured by enzyme linked immune sorbent assay before down regulation of ovaries and initiation of treatment protocol of ART. Failure of procedure was detected by beta human chorionic gonadotropin <25mIU/ml (non-pregnant) whereas females with levels >25mIU/ml and cardiac activity on trans-vaginal scan were declared pregnant.

**Results:**

Highest Kisspeptin and Leptin levels were seen in normal weight group (374.80 ± 185.08ng/L; 12.78 ± 6.8 pg/ml) respectively, yet the highest number of clinical pregnancy was observed in overweight group (42%).A strong correlation of Kisspeptin with Leptin (r=0.794, p=0.001) was observed in the overweight females.

**Conclusion:**

Leptin-Kisspeptin-fertility link is expressed by maximum number of clinical pregnancies in the female group that showed strongest relationship between serum Leptin and Kisspeptin levels, irrespective of their BMI.

## INTRODUCTION

A person’s body weight, fat content and overall metabolic state are pertinent modifiers for puberty and fertility.^1^ Metabolic stress in terms of over nutrition and under nutrition is frequently linked to reproductive disorders.^2^ The connection between energy balance and reproduction are linked with the discovery of adipocytokine however, our understanding of the neurobiological basis for this phenomenon is still incomplete.

Adipokines i.e. cytokine/hormones or enzymes secreted form adipose tissue are now considered as a missing link. A vast majority of adipokines do not act on the hypothalamic-pituitary-gonadal axis (HPG axis), yet their role in reproductive functions along with metabolic regulations has great potential. One such exceptional adipokine famous for its dualism is ‘Leptin’ which is secreted by both the adipose tissue and endometrial cells.^3^ Its role in obesity and reproductive regulation are well known.^4^ It appears to function as regulator of food quantity consumed relative to the amount of energy expended.^5^

Kisspeptin (KP), an RF-amide coded by the *KISS*1 gene is a major neuroendocrine regulator of reproduction, that intricacies between central, peripheral reproductive-neuro-endocrine axis.^6^,^7^ It plays a focal part in the control of the HPG axis neuronal activity (highly sensitive to the body energy stores and nutritional status) to trigger puberty as well as affects fertility.^7^,^8^ KP not only triggers onset of puberty but also relays information about body’s energy stores to the central nervous system by modulating negative and positive feedback of gonadal steroids.^9^ The expression of *KISS*1 is thus influenced by nutritional status where KP neurons function as a gatekeeper of reproductive function at pivotal times in the human lifespan, down-regulating fertility at times of physical strain such as over-exercise or weight loss.^10^

The Leptin-KP harmony lies in the source and target as in ‘Hypothalamic neurons’, where they cross talk with anorexigenic pro-opiomelanocortin and orexigenic neuropeptide Y neurons.^11^,^12^ Furthermore, presence of Leptin receptor (Ob-Rb) on KP neurons in the mouse arcuate nucleus,^13^ suggests a role for KP in mediating the metabolic signals of Leptin on the HPG axis. These interactions of metabolic and KP signaling in the hypothalamic networks are proven, yet little explanation on their existence in the periphery is available.^14^ Both Leptin and KP are linked to regulation of energy metabolism and reproduction;^15^ though, the interactions of these hormones with infertility and the treatment outcomes remain unclear. Being aware of the presence of peripheral receptors of Leptin and KP, we wanted to explore the association of serum Leptin, KP and Body Mass Index on success after assisted reproductive treatment (ART) after suppression of HPG axis.

## METHODS

A cross sectional study was carried from August 2014 till May 2016 after receiving ethical approval at Australian Concept Infertility Medical Centre, and Aga Khan University. The study group comprised of females with an age range of 25-37 year who had duration of unexplained infertility for more than two years. The decision for unexplained infertility was made following established protocol;^16^ that states that the diagnosis of unexplained infertility can be made only after excluding common causes of infertility using standard fertility investigations, which include semen analysis, assessment of ovulation, and tubal patency test. Females with known causes of infertility like polycystic ovaries, uterine fibroids, male factor and other metabolic disorders were excluded from the study. The study subjects were categorized as underweight (<18.5 kg/m^2^), normal weight (18.5–22.9 kg/m^2^), overweight (23.0 –24.9 kg/m^2^) and obese (≥25 kg/m^2^) based on the guidelines for South Asian population. Long protocol of down regulation of ovaries was achieved by sub cutaneous administration of 1 mg of subcutaneous Buserelin Acetate (Suprefact, Sanofi Aventis, Pakistan) in mid luteal phase of previous cycle. Down regulation of ovaries was confirmed by a trans vaginal scan (TVS) which showed a thin endometrial lining and quiet ovaries without any cyst and blood test with estradiol levels below 30pg/ml. Five millilitres of fasting blood sample was collected from each subject after down regulation of ovaries in order to estimate Serum KP and Leptin levels by commercially available ELISA Kit (Cat.No:YHB1811Hu; Cat. No ADI-900-028A) following the manufacturer’s protocol.

Controlled ovarian stimulation (COS) was carried out by using subcutaneous administration of recombinant FSH (r FSH) 50 IU preparation (Puregon registered; NV Organon, Oss, The Netherlands). The initiation dose was calculated on the basis of age of the subject, basal serum FSH concentrations, AFC and BMI. Dose was later adjusted by follicular tracking with TVS (7.5 MHz probe Aloka 500, Tokyo Japan) from the fifth day of COS on alternate days. COS was followed by, proper timing for hCG administration (ovulation induction), oocytes retrieval, followed by in vitro fertilization (IVF).(6) Failure of procedure was detected by beta human chorionic gonadotropin <25mIU/ml (non-pregnant) whereas females with levels >25mIU/ml and cardiac activity on trans-vaginal scan were declared pregnant.

Data was analysed using SPSS 21. Descriptive analysis of variables was expressed as mean ± standard deviation. Independent t test and one way analysis of variance (ANOVA) were used to compare continuous variable wherever applicable. Pearson correlation was applied to test the link between KP-Leptin and clinical pregnancy or BMI. Receiver operating curve was plotted to see the difference between groups. In all cases a p value of < 0.05 was considered as significant.

## RESULTS

Hormonal analysis of one hundred and thirty-two infertile females was included in this study. Overall, the mean age and BMI of the study population was 32.12 ± 4.7 year and 24.19 ± 3.56 kg/m^2^ respectively. Table-I segregates the study cohort on the bases of pregnancy outcomes that showed no difference in the age, BMI, follicle stimulating hormone (FSH) and luteinizing hormone (LH) whereas the variation in levels of KP, Leptin and estradiol was observed to be highly significant (p= 0.001, 0.025 & 0.001 respectively).

**Table-I:** Descriptive analyses of variables on the basis of Pregnancy outcomes.

Variable	Clinical pregnancy n=31 (Group A) Mean ± SD	No pregnancy n=101 (Group B) Mean ± SD
Age (year)	32.31 ± 5.1	32.06 ± 4.5
BMI (kg/m^2^)	23.11 ± 3.33*	24.60 ± 3.57
Kisspeptin (ng/L)	369.87 ± 138.47*	221.39 ± 96.27
Leptin (ng/ml)	11.57 ± 6.2**	8.80 ± 5.2
FSH (IU/L)	6.31 ± 0.89	6.14 ± 0.87
LH (IU/L)	6.04 ± 1.3	5.16 ± 1.05
Estradiol pg/ml	250.28 ± 68.9**	150.4± 36.02
No. of fertilized oocytes	6.61 ± 1.15*	5.79 ± 1.2
No. of transferred embryo	1.80 ± 0.62	1.60 ± 0.57
Endometrial thickness (mm)	11.30 ± 2.1**	6.9 ± 2.8

Where: LH (luteinizing hormone), FSH (follicular stimulating hormone), BMI (body mass index).

* T-test was applied to calculate difference between groups. p-value of <0.05,

** p-value of <0.01.

Levels of serum KP and Leptin were observed to be highest in the normal weight group (p = 0.001 and 0.002 respectively). Regarding serum KP, post-hoc analysis reported a significant difference in the normal weight group in comparison to overweight and obese population (p<0.01). Likewise, serum Leptin was also found to be significantly higher in the normal weight group in comparison to other three groups. However, while analyzing the implantation rate of embryo, the overweight group reported maximum success rate followed by underweight group (Table-II).

**Table-II:** Descriptive statistics of study population categorized on the basis of BMI.

Variable	Underweight (<18 kg/m^2^) Mean ± SD n = 22	Normal weight (18-23 kg/m^2^) Mean ± SD n =37	Overweight (23-25 kg/m^2^) Mean ± SD n =24	Obese (> 25 kg/m^2^) Mean ± SD n = 49
Age (year)	31.37 ± 3.9	30.45 ± 4.7	31.79 ± 4.6	32.36 ± 3.5
Kisspeptin(ng/L)	271.03 ± 180	374.80 ± 185.08*	245.15 ± 96.14	196.03 ± 83.08
Leptin (ng/ml)	7.29 ± 1.5	12.78 ± 6.8*	9.08 ± 5.77	8.12 ± 6.16
FSH (IU/L)	6.53 ± 0.74	6.34 ± 1.02	5.94 ± 0.97	6.12 ± 0.73
LH (IU/L)	4.91 ± 0.97	5.58 ± 1.21	6.02 ± 1.06	4.97 ± 1.19
Estradiol (pg/ml)	214 ± 45.01	180 ± 69.40	221.88 ± 75.72	150.23 ± 64.82
No. of oocytes fertilized	5.75 ± 1.2	5.86 ± 1.58	6.29 ± 1.26	6.05 ± 1.08
No. of transferred embryo	1.41 ± 0.51	1.56 ± 0.55	1.66 ± 0.56	1.7 ± 0.62
Implantation rate	29.17 ± 15.01	24.77 ± 6.86	34.03 ± 23.81	16.67 ± 14.47
Clinical pregnancy	37 %	29 %	42 %	19 %
No pregnancy	63 %	71 %	58 %	81 %

Where: LH (luteinizing hormone), FSH (follicular stimulating hormone), BMI (body mass index) Data is expressed as Mean ± SD, or percentages.

* p<0.05)

**Fig.1:**
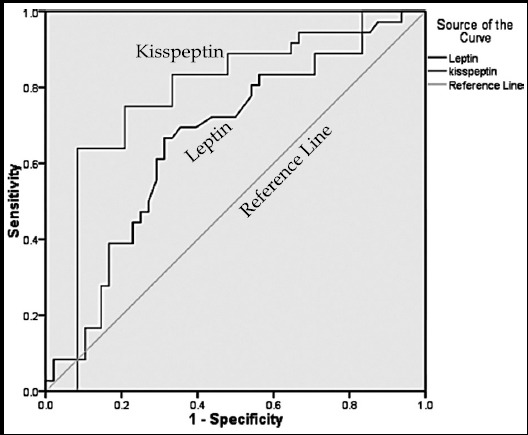
Receiver operating characteristic (ROC) curve for Kisspeptin and Leptin, determining area under the curve (AUC) in the group that attained clinical pregnancy (0.786; 95% CI 0.697-0.876) as compared to Leptin (0.663; 95% CI 0.560-0.765).

Next, serum KP and Leptin were analyzed by receiver operating characteristic (ROC) curve in the females who achieved clinical pregnancy to compare their relevance to positive outcomes (Fig.1). Area under the curve (AUC) for KP was found greater (AUC= 0.786; 95% CI 0.697-0.876) as compared to Leptin (AUC=0.663; 95% CI 0.560-0.765).

In order to gain insight, we studied correlation of serum KP with the levels of Leptin in clinical pregnancy group as well as no conception group. We observed a highly significant moderately positive correlation of KP-Lpetin in the clinical pregnancy group (r=0.540; p<0.001). On the other hand non-significant correlation was seen in the non-pregnancy group (r=0.110; p=0.287). Interestingly, overweight (r=0.794; p=0.001) and underweight (r=0.841; p=0.001) group, that reported highest clinical pregnancy were found to have better correlation among the two hormones as compared to normal weight group (r=0.233; p=0.166) and obese (r=0.082; p=0.538).

## DISCUSSION

High prevalence of infertility and the related issues has directed couples towards assisted reproductive techniques (ART) in our group of population.^17^,^18^ Human reproductive function is influenced by a balanced relationship between key hormones associated with metabolic status and reproduction. Low KP levels were observed in our obese cohort that can be compared with studies done in obese poly cystic and pre- eclamptic females.^19^,^20^

Observational studies have demonstrated that states of Leptin excess, deficiency, or resistance can be associated with abnormal reproductive function. In our study the high circulatory levels of Leptin in clinical pregnancy in comparison to non-pregnant group, has been observed by previous researchers where circulatory levels were related to repeated spontaneous abortions and higher levels with superior implantation success rates.^21^,^22^

Thus, we can speculate that Leptin-KP interplay in the central nervous system is responsible for reproductive deficits associated with Leptin-deficient states due to diminished expression of *Kiss1*. KP signaling at the same time is also shown to influence body weight, energy expenditure, and glucose homeostasis in a sexually dimorphic and partially sex steroid–independent manner.^23^,^24^

As far as BMI is concerned, a cut off value of less than 26 kg/m^2^ was associated with positive pregnancy outcome in our population.^25^ We also observed maximum clinical pregnancies (42%) in females with BMI 23-24.9 kg/m.^2^ As far as interplay of KP- Leptin and BMI is concerned, the group although had low Leptin and KP levels yet a strong significant positive correlation was observed. These facts support our notion that correlation between hormones is more critical than their mere normal level. This observation in the present study supports the role of Leptin-KP on ovarian and endometrial functions which is reinforced by the presence of Leptin receptors on these tissues. Despite these promising results, our study is limited in a way that we were unable to collect multi centre data. Moreover, Leptin concentration should ideally be performed in follicular fluid in addition to the serum level.

## CONCLUSION

A greater reproductive potential was observed in the group of females that exhibited strong association of Leptin with KP. Whether this link is predominantly permissive for diagnostic value, further in-depth exploration of the interplay of “Leptin-Obesity-Kisspeptin-Fertility” at molecular levels is expected to shed light in future.
